# Analysis of clinical studies on clozapine from 2012-2022 

**DOI:** 10.1007/s00210-024-03209-1

**Published:** 2024-06-25

**Authors:** Anton Freibüchler, Roland Seifert

**Affiliations:** Institute of Pharmacology, Hannover Medical School, Carl-Neuberg-Straße 1, Hannover, 30625 Germany

**Keywords:** Clozapine, Schizophrenia, Schizoaffective disorder

## Abstract

Clozapine has been considered the “gold standard” in the treatment of schizophrenia for many years. Clozapine has a superior effect, particularly in the treatment of negative symptoms and suicidal behaviour. However, due to its numerous adverse reactions, clozapine is mainly used for treatment-resistant schizophrenia. The aim of this paper is to analyze the results of clinical studies on clozapine from 2012-2022. PubMed was used as the database. Sixty-four studies were included and categorised by topic. The pharmacokinetic properties of clozapine tablets and a clozapine suspension solution did not differ markedly. Clozapine was superior to olanzapine and risperidone in reducing aggression and depression. A long-term study showed that metabolic parameters changed comparably with olanzapine and clozapine after 8 years. Risperidone and ziprasidone can be used as an alternative to clozapine. Scopolamine, atropine drops, and metoclopramide are effective in the treatment of clozapine-induced hypersalivation. Eight drugs, including liraglutide, exenatide, metformin, and orlistat, are potentially effective in the treatment of clozapine-induced weight gain. Ziprasidone, haloperidol, and aripiprazole showed a positive effect on symptoms when added to clozapine. No investigated drug was superior to clozapine for the treatment of schizophrenia. Ziprasidone and risperidone can also be used well for the treatment of schizophrenia. In the treatment of clozapine-induced hypersalivation and weight gain, some drugs proved to be effective.

## Introduction

**Fig. 1:**
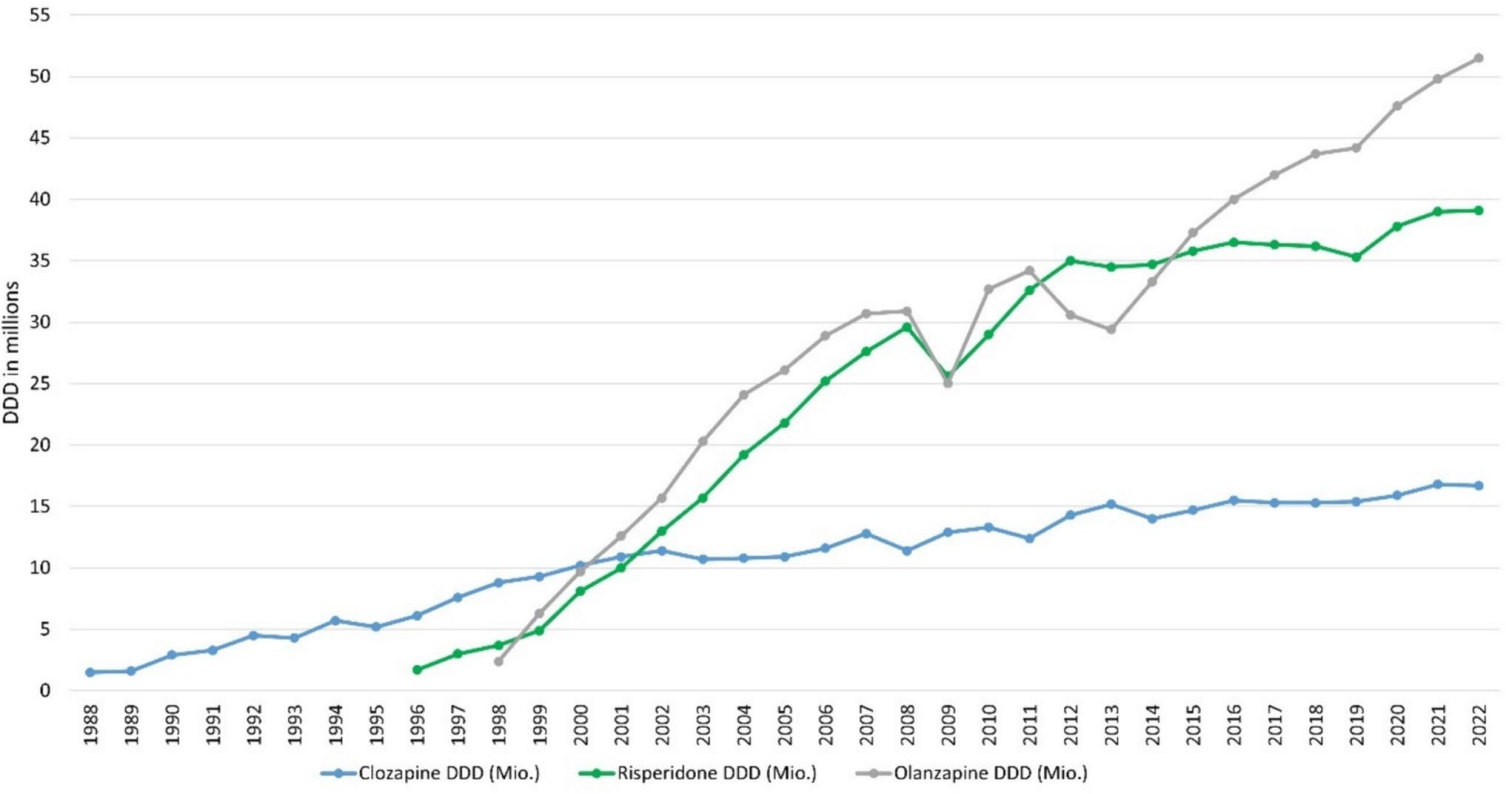
DDD in millions for clozapine, risperidone, and olanzapine for 1988–2022 in Germany. Data were taken from the Drug Prescription Report (Arzneiverordnungsreport, AVR) of the statutory health insurance

**Fig. 2:**
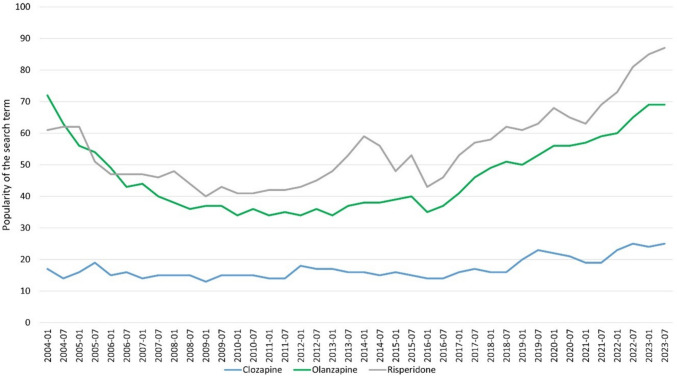
Development of search interest on Google for clozapine, risperidone, and olanzapine (https://trends.google.com/trends/explore?date=all&q=%2Fm%2F0b_th,%2Fm%2F019bkd,%2Fm%2F019bht&hl=de, accessed 13 December 2023)

**Fig. 3:**
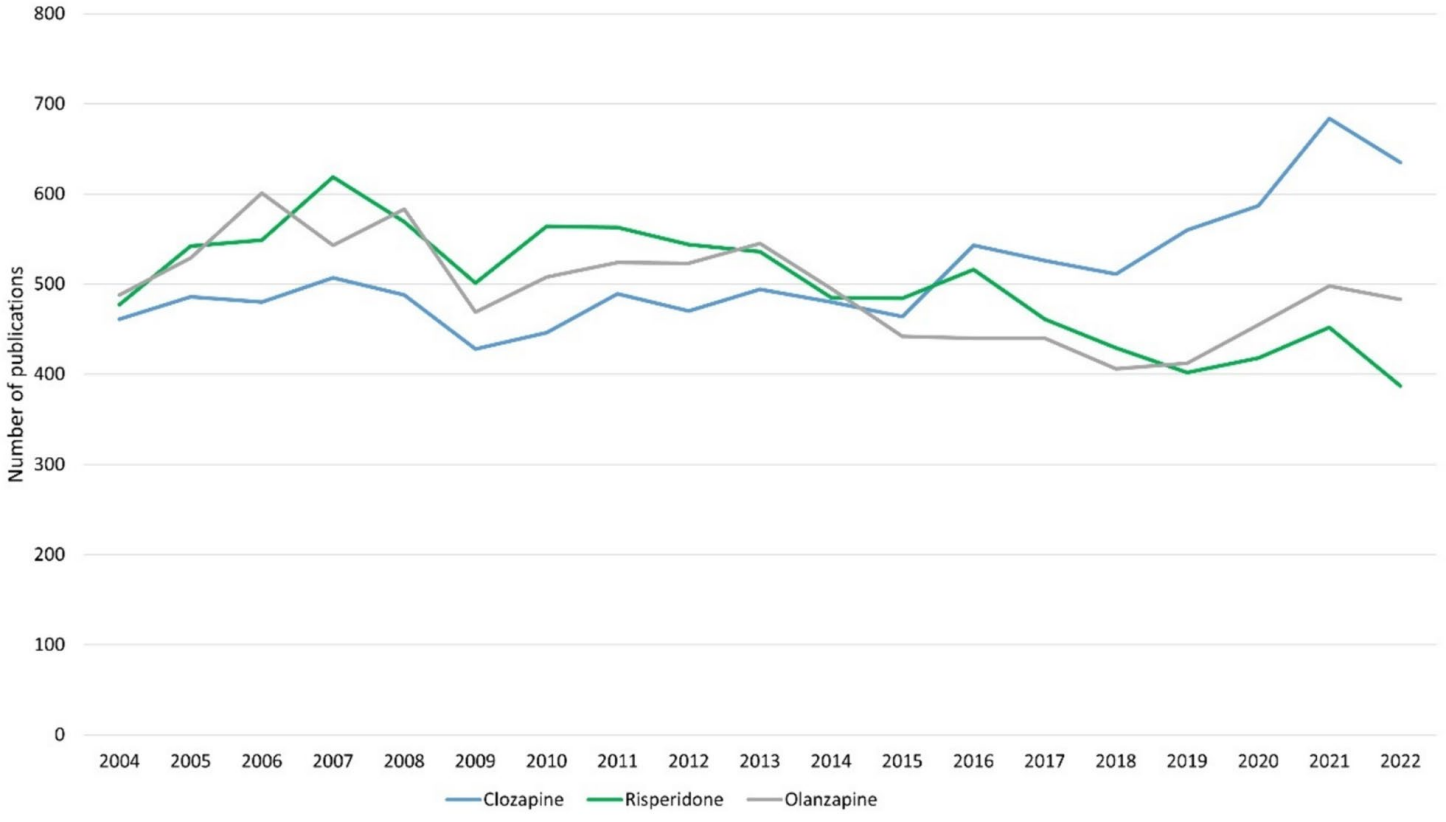
Publications on PubMed on clozapine, risperidone, and olanzapine, 2004–2022 (https://pubmed.ncbi.nlm.nih.gov/?term=Clozapin&filter=years.2004-2022, accessed 03 March 2024; https://pubmed.ncbi.nlm.nih.gov/?term=Risperidon&filter=years.2004-2022, accessed 03 March 2024; https://pubmed.ncbi.nlm.nih.gov/?term=Olanzapin&filter=years.2004-2022, accessed 03 March 2024)

The worldwide prevalence of schizophrenia is around 1% and affects women just as much as men. People who develop schizophrenia often suffer from very pronounced symptoms. These include hallucinations, delusions, apathy, loss of interest, and social withdrawal (Seifert, Roland: Basiswissen Pharmakologie, 2^nd^ edition, Germany, Springer, 2021, page 404). It is therefore important to develop a therapy that is as efficient as possible but also has as few adverse drug reactions as possible. Clozapine has been considered the “gold standard” for the treatment of schizophrenia for many years and its efficacy has been proven by numerous studies (Mizuno et al. 2020; Stroup et al. 2016). Nevertheless, clozapine is primarily used for treatment resistance but rarely as a first-line therapy (Chakos et al. 2001). This is partly due to the rare but dangerous adverse drug reaction of agranulocytosis. Fear of agranulocytosis has led to an increase in the use of alternative drugs, which, however, have generally proved less effective (Seifert, Roland: Basiswissen Pharmakologie, 2^nd^ edition, Germany, Springer, 2021, page 411). In Germany, clozapine has shown a significantly lower increase in prescription figures than risperidone and olanzapine since 1996. While only 2.4 million defined daily dose (DDD) of olanzapine were prescribed in 1998, this figure rose to 51.5 million DDD by 2022. During this period, the prescribed DDD of clozapine rose from just 8.8 million DDD to 16.7 million DDD (Fig. 1). However, this could also be due to the fact that olanzapine and risperidone are used for other diseases than schizophrenia: olanzapine and risperidone for manic episodes and bipolar disorders, risperidone additionally for Alzheimer’s dementia patients who display aggressive behaviour (https://www.gelbe-liste.de/wirkstoffe/Olanzapin_26411#Anwendung, last accessed 24.02.2024; https://www.gelbe-liste.de/wirkstoffe/Risperidon_20541#Anwendung, last accessed 24.02.2024). Google search trends for clozapine are less pronounced than those of  olanzapine and risperidone. The latter two drugs have seen a noticeable increase in Google searches since 2007, but almost none was noticed for clozapine (Fig. 2). The interest in clozapine in the general public therefore appears to be generally low. In marked contrast, there is no evidence of low interest in clozapine in PubMed. Rather, the number of publications on clozapine is increasing while the number of studies on olanzapine and risperidone rather decreases (Fig. 3). The pharmacological and psychiatric community is therefore interested in research on clozapine.

The aim of this study is to analyse the clinical clozapine studies of the last 10 years (2012–2022) in more detail to gain new insights into the treatment of schizophrenia and the use of clozapine. Particular attention will be paid to drugs that could be combined with clozapine.

## Material and methods

### Study selection

**Fig. 4:**
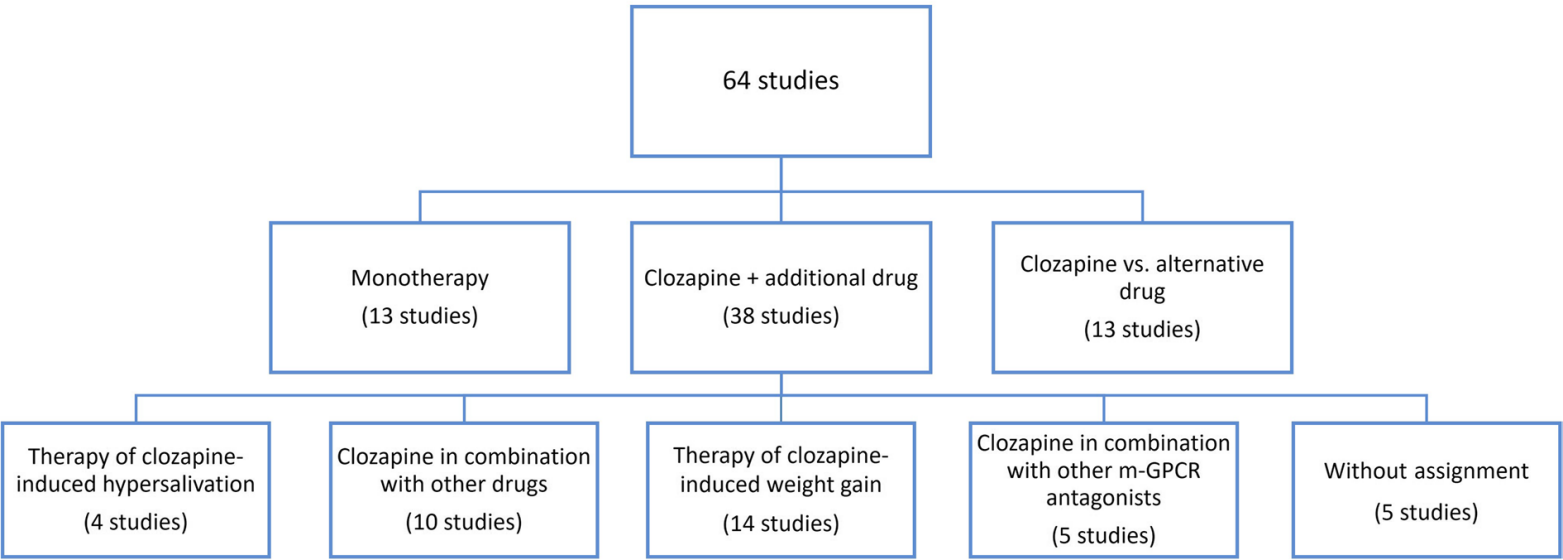
Overview of the assignment of studies to subgroups

When compiling the literature for this publication, the clozapine studies of the last 10 years were searched (2012–2022). The database selection was limited to PubMed. During this period, there were a total of 90 studies, 26 of which did not meet the inclusion criteria. Only studies that dealt with clozapine therapy and had already been completed were included. Sixty-four studies met these criteria (Fig. 4). Due to the heterogeneous subject matter of these studies, three subgroups were formed to compare the study results with each other. The subgroups are made up as follows: clozapine monotherapy, clozapine vs. other “antipsychotic” drugs and clozapine + additional drug. The group “clozapine monotherapy” includes all studies, in which patients received clozapine only, no matter which endpoint was examined. The group “clozapine + additional drug” was further subdivided into five subgroups: therapy of clozapine-induced hypersalivation, therapy of clozapine-induced weight gain, clozapine in combination with another drug, clozapine in combination with another m-GPCR antagonist. Five studies remained unclassified (Fig. 4). We replaced the colloquial and clinically used term "antipsychotic" by the term  “m-GPCR antagonist (antagonist at multiple G-protein-coupled receptors)”. This means that the drug is more precisely assigned to its molecular mechanism, which has two advantages. It is easier to recognise that a drug acting on multiple receptors has also multiple indications and multiple adverse effects, and that the patient is not prescribed an “antipsychotic”, which can lead to confusion, as the indications of these drugs are no longer limited to mental illnesses (Seifert and Schirmer 2020; Seifert and Schirmer 2021). The studies were analysed based on various analysis parameters: study type, type of randomisation, initial diagnosis, number of participants, endpoints, age range, mean age, study duration, mean clozapine dose, study country, place of publication, and financial support. The results were summarised in tables.

### Data analysis

The tables with the analysis results were graphically presented using Microsoft PowerPoint. Furthermore, the study results of the alternative/additional medication were summarised in tabular form. A traffic light system was introduced to provide a quick overview. In the table clozapine vs. another drug, the drug that was more effective was always highlighted in green. If both were effective, both were marked green. In the tables on additional medications, the additional drug was labelled green if it had a positive effect on treatment with clozapine, yellow if the results were inconclusive or controversial, and red if no benefit could be found for the therapy. The study results were also compared with results from four previous reviews.

## Results and discussion

### Overview of all studies

#### Initial diagnosis of the participants

**Fig. 5:**
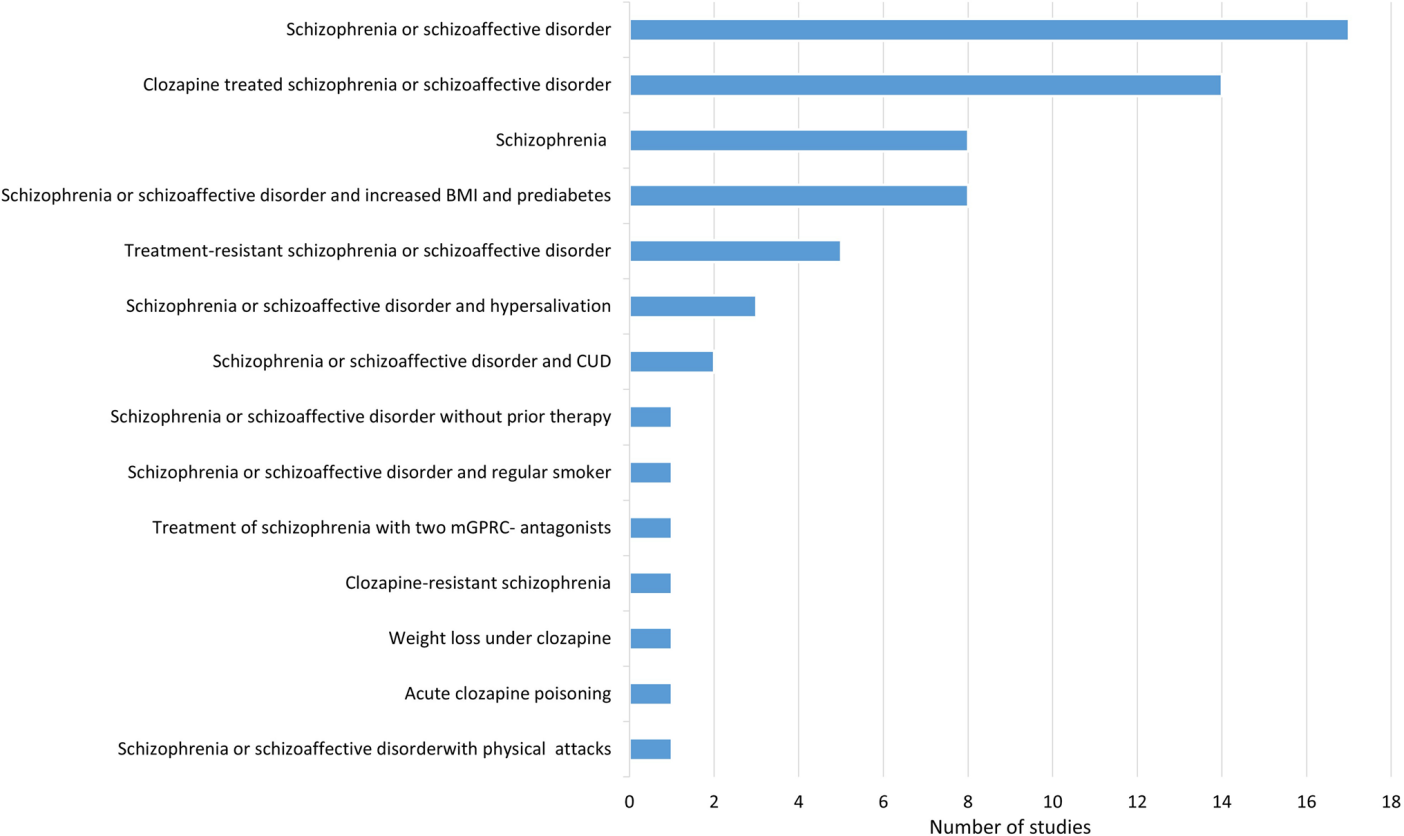
Initial diagnosis of the participants (s, schizophrenia; sd, schizoaffective disorder)

Most of the study participants suffered from schizophrenia or schizoaffective disorder (Fig. 5). Additional criteria for the enrollment of patients in the study were adverse effects. In most of these studies, add-on drugs to clozapine were tested to examine their influence on the treatment of schizophrenia, but also on the adverse effects. Some of the studies were conducted on patients with treatment-resistant schizophrenia. In these cases, the efficacy of clozapine was tested in comparison with other drugs. Possible additional medications were also tested here. Studies were also carried out on patients with schizophrenia who had not yet received any treatment. The aim was to test the efficacy of clozapine as a first-line therapy (Fig. 5).

#### Study country

**Fig. 6:**
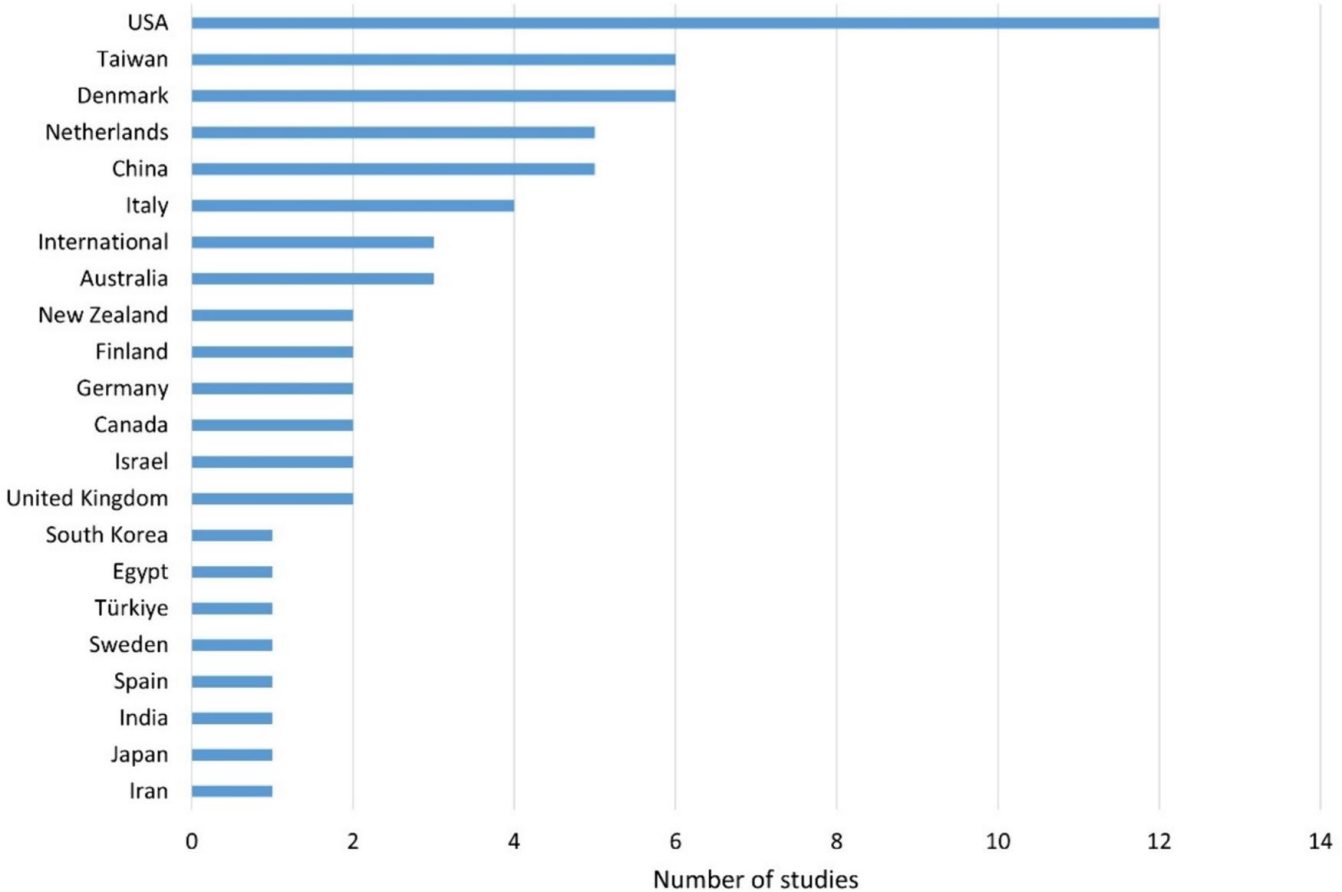
Overview of the countries where the studies were conducted

A total of 21 different countries are represented, nine of which are European. Twelve of the studies come from the USA, but also six each from Taiwan and Denmark (Fig. 6). Taiwan has traditionally conducted a great deal of research in the field of psychiatry. The additional medications in the studies from Denmark are on sertindole and liraglutide. Sertindole is produced by H. Lundbeck and liraglutide by Novo Nordisk. Both companies are located in Denmark and financed the respective studies.

#### Duration of the studies

**Fig. 7:**
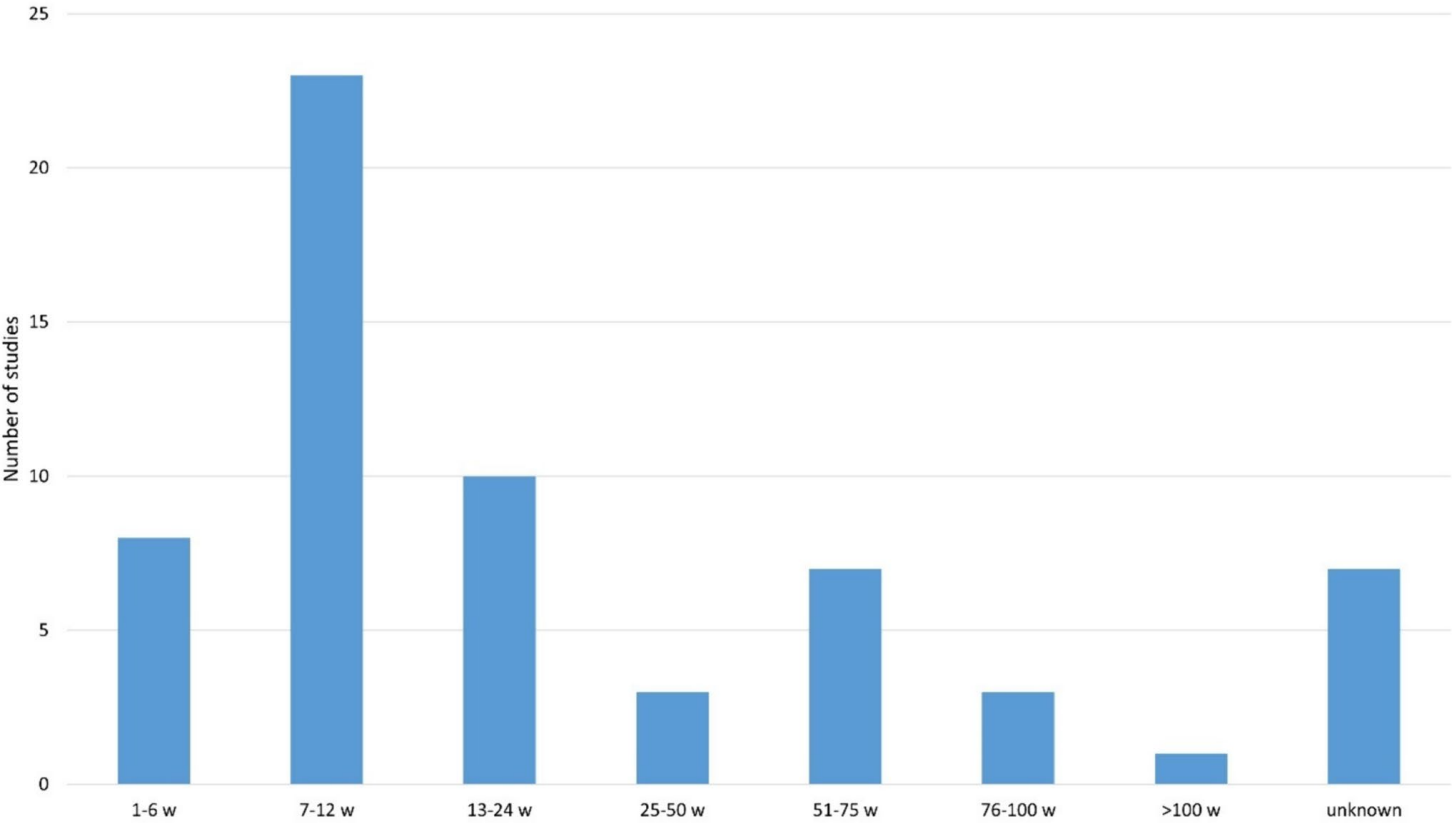
Overview of the duration of the studies

Most studies had a study duration of 1–24 weeks, 23 of which were in the 7–12 week range (Fig. 7). Particularly in the group of additional medication, only 6/38 studies were longer than 26 weeks and of these six, two are controls after 1 year from previous studies. This means that the additional therapy was not continued after the study period; it was just checked how the analysed parameters had developed after 1 year. A single study was longer than 2 years, namely 8 years. Here, the long-term effects of clozapine on metabolic parameters were analysed in comparison with olanzapine. The duration of the studies must be viewed critically. Schizophrenia patients often require treatment for several years or even a lifetime, so short observation periods cannot provide any information about how the symptoms present after a few years (https://www.neurologen-und-psychiater-im-netz.org/psychiatrie-psychosomatik-psychotherapie/stoerungen-erkrankungen/schizophrenie-und-schizophrene-psychosen/therapie/#:~:text=In%20schizophrenia%2Dpatients%20with%20several%20chronic%20courses%20even%20permanent; accessed 05 December 2023). The same applies to possible additional medications, which must also be administered over several years to maintain their effectiveness. Exenatide is a good example of this. Patients who were successfully treated with exenatide for 24 weeks returned to their initial weight after discontinuation (Siskind et al. 2020; Siskind et al. 2018a). From this result, it can only be concluded that exenatide can reduce body weight over the tested period. This shows that the study periods were often too short. Further studies are needed to test the long-term efficacy of some of the additional medications.

#### Financing of the studies

**Fig. 8:**
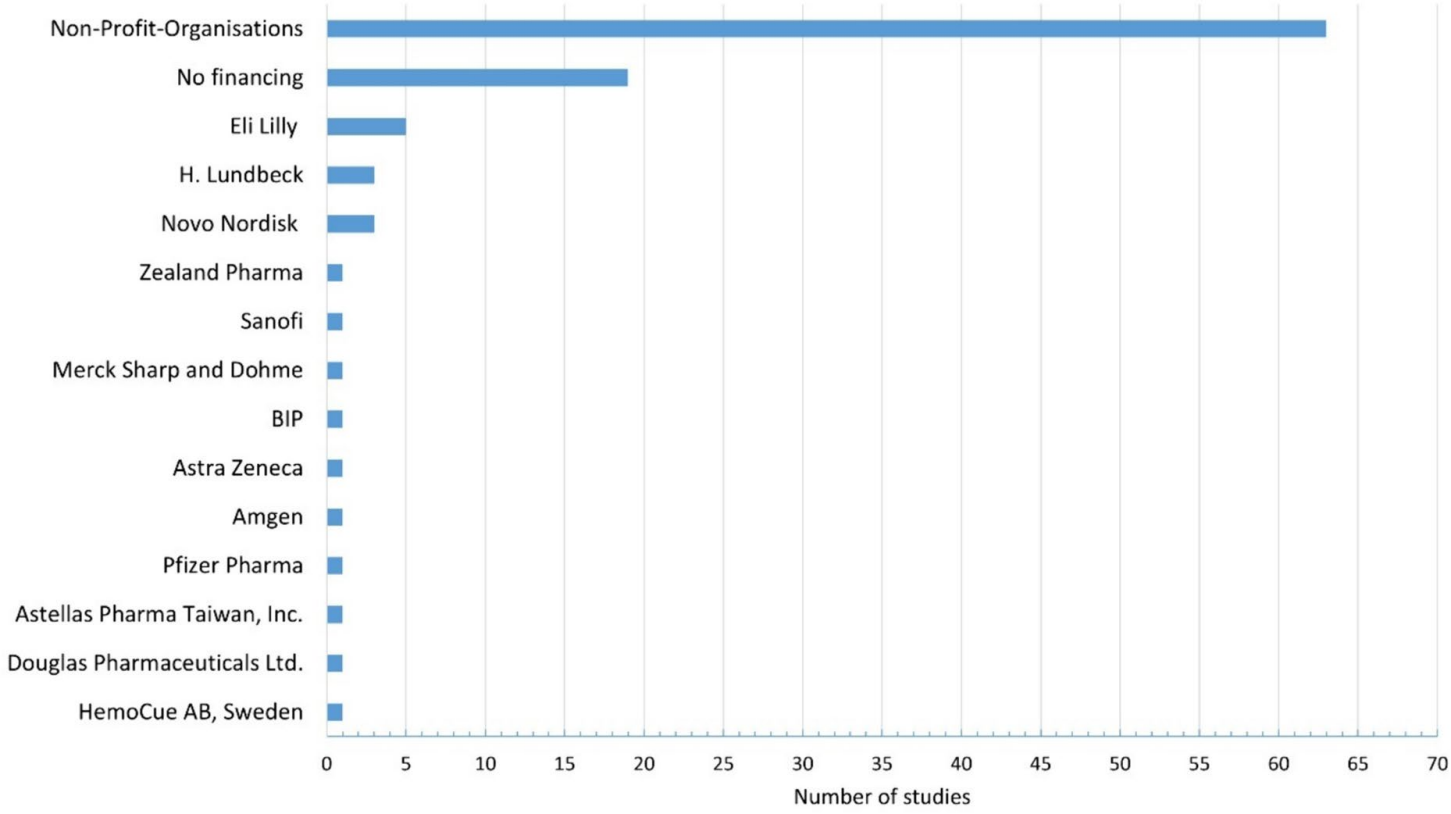
Overview of financial support for studies (BIP, Boehringer Ingelheim Pharmaceuticals)

Non-profit organisations accounted for most funding sources for the studies (Fig. 8). Public grant agencies, universities, and foundations with no commercial interest were summarised under this umbrella term. Thirty percent (N = 19) of the studies were not funded. Among the pharmaceutical companies that funded a study, there is none that produces clozapine. If a study was financially supported by a company, it were probably the additional drugs that were of commercial interest to the companies. Liraglutide from Novo Nordisk, sertindole from H. Lundbeck, and zotepine from Astellas Pharma, Taiwan Inc., are three examples. The patent for clozapine had been expired for many years, and therefore very cheap generics are available. Companies have little interest in clozapine research, as this would not be associated with high profits. However, effective add-on medications to clozapine could be of great financial interest, as clozapine must be given over a long period of time. Another reason for the lack of interest of pharmaceutical companies in clozapine could be that the efficacy of clozapine has been proven for many years, and therefore no further studies are needed (Seifert, Roland: Basiswissen Pharmakologie, 2^nd^ edition, Germany, Springer, 2021, page 411).

#### Monotherapy

A total of 13 studies were assigned to this subgroup, nine of which will be presented here. The other four examined topics were too heterogeneous.

Three clinical trials on the effects of clozapine on the heart have shown that even small doses lead to a subclinical decrease in left ventricular function. This applies to both diastolic and systolic function. Biventricular dysfunction was even observed in 10/38 participants. CRP was also elevated, although the plasma concentration of troponin-I and natriuretic peptide remained within the normal range (Curto et al. 2015; Rostagno et al. 2012). It is, therefore, important to monitor cardiac function during clozapine therapy, especially in patients with pre-existing cardiovascular conditions. However, this effect could be utilised to determine whether a patient is responding to clozapine (Kim et al. 2013). 

TDM should keep an eye on metabolic parameters and on agranulocytosis. If agranulocytosis occurs, clozapine must be discontinued immediately, and the patient monitored closely. Treatment with granulocyte colony-stimulating factor (G-CSF) appears to improve the outcome of patients (https://www.gelbe-liste.de/krankheiten/agranulozytose#:~:text=Agranulocytosis%20is%20a%20sepsis%20when%20severe%20and%20untreated%20most%20of%20the%20time; accessed 05 December 2023). This makes it all the more important to develop a good setting for TDM. Capillary blood sampling was better accepted by patients than venous blood sampling (Bogers et al. 2015). The time of blood sampling can be chosen between 10 and 14 h after clozapine administration without affecting the clozapine plasma concentration (Jakobsen et al. 2017). The type of administration is also part of the setting of a good therapy, as not all patients cope well with tablets. A clozapine 50 mg/ml suspension solution (Douglas Pharmaceuticals, Auckland, New Zealand) behaved on a par with Clozaril® in terms of pharmacokinetic properties (Glue et al. 2012). This was true under food intake and fasting conditions, so the filling state of the stomach appears to have no influence on absorption. These studies make it possible to create an individualised setting for treatment with clozapine. 

Genotyping of patients could provide information in advance whether a patient responds to clozapine. Certain gene polymorphisms are associated with a better response to clozapine than others. The gene ITIH3 rs2535629 was analysed (Brandl et al. 2016). The same applies to the side effect of weight gain. The GABAA2 gene of the patients (*N* = 160) was analyzed. This could be used to filter out patients who are likely to gain a lot of weight on clozapine before starting treatment (Zai et al. 2015). For these patients, weight-reducing therapy could be started in good time. Certain gene sequences have also previously been identified as risk alleles for mental illnesses, particularly schizophrenia (Anonymous 2013). However, it remains questionable whether the benefits justify the effort and whether this can be realised financially. 

#### Clozapine vs. other drugs

None of the eight alternative treatment options was superior to clozapine in reducing the symptoms of schizophrenia (Table 1). Risperidone, ziprasidone, and ECT showed comparable efficacy to clozapine, while olanzapine, haloperidol, and quetiapine were inferior. In one study, clozapine improved the positive symptoms of patients better than ziprasidone, but it was also associated with more adverse drug reactions, so patients accepted ziprasidone better (Schnell et al. 2014). The effect on metabolic parameters and improving PANSS was significantly better with ziprasidone in the other study. In this study, patients who had been taking clozapine for 2 years were switched to ziprasidone (Li et al. 2013). Ziprasidone could be an alternative to clozapine.

Risperidone is also an alternative to clozapine. Both drugs led to promising results both as initial therapy for schizophrenia and in refractory patients, with clozapine being superior to risperidone. An earlier study has already shown that clozapine is superior to olanzapine, risperidone, and quetiapine in refractory schizophrenia (McEvoy et al. 2006). The probability of discontinuing treatment was significantly lower with clozapine (Sanz-Fuentenebro et al. 2013; Schooler et al. 2016). In patients with CUD in addition to schizophrenia, clozapine significantly improved the PANSS compared to risperidone (Machielsen et al. 2014).

Olanzapine was most frequently compared with clozapine (five studies; Table 1). Clozapine was superior to olanzapine in reducing aggressive behaviour, with olanzapine being more effective than haloperidol. The full potential of the anti-aggressive effect of clozapine may even have been underestimated in this study. Clozapine had to be increased slowly for clinical reasons, so that only a short period of time under the full dosage could be assessed. This was not the case with the other two drugs (Krakowski et al. 2021). These findings are supported by previous results that demonstrated the superiority of the anti-aggressive efficacy of clozapine compared with olanzapine, risperidone, and haloperidol (Citrome et al. 2001). Olanzapine led to a better quality of sleep, but also to a more frequent manifestation of diabetes than clozapine. The other metabolic parameters changed similarly with both drugs. This was established in a long-term therapy in which the patients were regularly monitored over a period of 8 years (Feng and Melkersson 2012; Kluge et al. 2014).

Zotepine is similar to clozapine in terms of structure and pharmacological profile. Switching refractory schizophrenia patients from clozapine to zotepine should be done with caution. Zotepine was associated with more adverse drug reactions and was less effective than clozapine (Lin et al. 2013).

In addition to a drug alternative to clozapine, the effectiveness of electroconvulsive therapy was investigated. The primary endpoint was the PANSS. Both therapy options significantly reduced the PANSS, with ECT being significantly better than clozapine. ECT led to an improvement in cognitive deficits (Mishra et al. 2022).

**Table 1:**
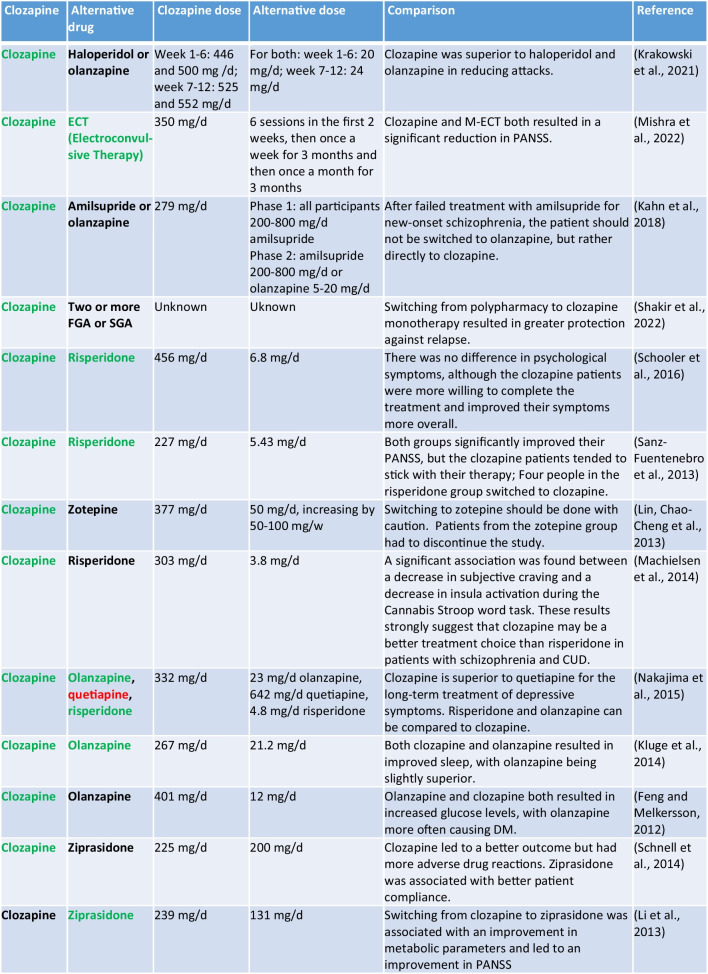
Summary of drugs compared with clozapine (green, better result than the other drug; if both drugs are green, both drugs have led to similar results)

### Add-on therapy to clozapine

#### Clozapine-induced hypersalivation

Clozapine-induced hypersalivation is a common adverse drug reactions. Clozapine antagonises the muscarinic receptors, which REDUCES salivation (https://www.gelbe-liste.de/wirkstoffe/Clozapin_10017#Nebenwirkungen, accessed 20 May 2024.). This is also a common adverse drug reaction. The mechanism of action for hypersalivation is not exactly known (Praharaj et al. 2006). Three drugs were tested for the treatment of clozapine-induced hypersalivation. As shown in Table 2, 600 μg/day of atropine drops and 10 mg/day of metoclopramide were effective in the treatment of hypersalivation (Kreinin et al. 2016; Mubaslat and Lambert 2020). If there was no response, metoclopramide was increased to up to 30 mg/day, which significantly reduced salivation. Like atropine drops, metoclopramide was well tolerated. Only one participant experienced increased salivation when taking atropine drops (Kreinin et al. 2016; Mubaslat and Lambert 2020). The third drug tested was scopolamine in two different dosage forms, as an ointment and as scopolamine hydrobromide. The ointment proved ineffective, but 0.3 mg/day scopolamine hydrobromide proved effective (Segev et al. 2019; Takeuchi et al. 2017). This is also the reason why scopolamine is highlighted in yellow in Table 2. In summary, three of the four drugs proved to be significantly effective in the treatment of clozapine-induced hypersalivation, making it possible for the practitioner to plan an individualised therapy for the patient. 

**Table 2:**
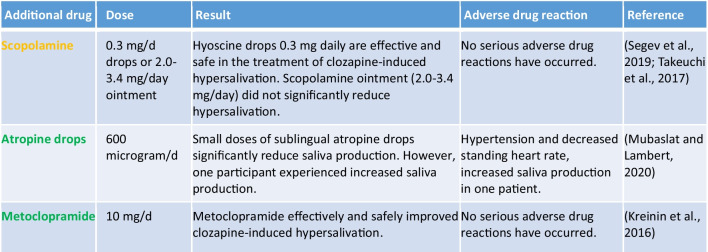
Overview of drugs for the treatment of clozapine-induced hypersalivation (green, the drug has a positive influence on hypersalivation; yellow, the influence on hypersalivation is not entirely clear; red, the drug has no influence on hypersalivation)

#### Clozapine-induced weight gain

Patients on clozapine very often gain a lot of weight (https://www.gelbe-liste.de/wirkstoffe/Clozapin_10017#Nebenwirkungen, accessed 20 May 2024). Nine drugs were analysed in 14 studies with regard to a possible treatment of this adverse drug reaction. Eight of the nine drugs led to a significant reduction in body weight. These are marked in green in Table 3. Only telmisartan was ineffective (Fan et al. 2019). Exenatide, liraglutide, metformin, orlistat, fluvoxamine (Lu et al. 2018), aripiprazole (Fan et al. 2013), and betahistine (Smith et al. 2018) all significantly reduced weight (Table 3). Liraglutide showed no additional effect on the bone turnover markers CTX and P1NP (Maagensen et al. 2021). Omega-3 fatty acids showed moderate effects on weight (Behdani et al. 2018). Orlistat significantly reduced weight and clozapine-induced constipation (Chukhin et al. 2013). The positive effect on weight of orlistat was described in a previous study. However, this only applied to male patients (Joffe et al. 2008). The symptoms of schizophrenia were also deteriorated by orlistat (Chukhin et al. 2016). Orlistat appears to be a promising drug for the treatment of clozapine-induced weight gain, particularly in male patients, and should be tested in further long-term studies. Liraglutide and exenatide significantly reduced weight during the treatment phase. One year after discontinuation of both drugs, the exenatide patients returned to their initial weight. In the liraglutide patients, the weight loss was maintained, but the metabolic parameters (plasma glucose, HbA1c, lipids, C peptide) deteriorated again (Siskind et al. 2020; Svensson et al. 2019). The same applies to metformin, which led to significant improvements in body weight and metabolic parameters at various doses (500 mg/day, 1000 mg/day, and 1500 mg/day). Twenty-four weeks after the end of therapy, all parameters returned to the initial values. In addition, certain genes appear to have an influence on metformin therapy. A significant reduction in insulin levels was observed in TMEM18 minor allele carriers. Sixty percent of TMEM18 and 40% of GNPDA2 minor allele carriers lost more than 7% of their body weight as a result of metformin treatment (Chen et al. 2013; Chen et al. 2015; Chiu et al. 2016). Metformin, liraglutide, and exenatide were highlighted in yellow because the positive effects did not persist after the end of treatment. Liraglutide, metformin, and exenatide showed potential for long-term therapy. These examples make it clear once again that there is a lack of further long-term studies on the treatment of adverse drug reactions. Only with these can a statement be made as to whether the drugs are suitable for long-term therapy with clozapine. 

**Table 3:**
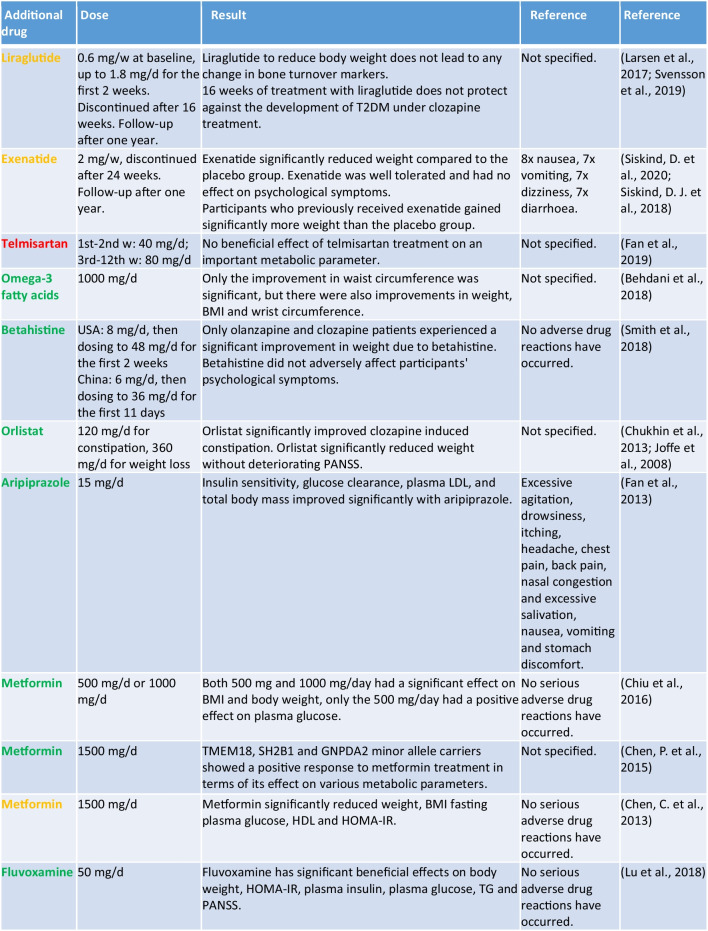
Overview of drugs for the treatment of clozapine induced weight gain (green, the drug leads to an improvement in body weight and metabolic parameters; yellow, the influence on weight and metabolic parameters is not yet clear; red, the drug does not lead to an improvement in weight or metabolic parameters)

#### Combination of clozapine with another drug

This subgroup summarises eight studies. These drugs are otherwise not classically indicated for schizophrenia. Minocycline is an antibacterial drug from the tetracycline group, memantine antagonises the glutamatergic NMDA receptors and is used in the treatment of moderate to severe dementia of the Alzheimer’s type (https://www.gelbe-liste.de/wirkstoffe/Memantin_2063, last accessed 06 December 2023), etc. As shown in Table 4, eight drugs were tested as adjuncts to clozapine, five of which showed positive effects on clozapine therapy. Minocycline increased clozapine plasma concentrations, but the results were not significant (Wehring et al. 2018). However, minocycline had a significant positive effect on the BPRS anxiety/depression factor and showed a significant improvement in working memory in participants in a post hoc analysis (Kelly et al. 2015).

**Table 4:**
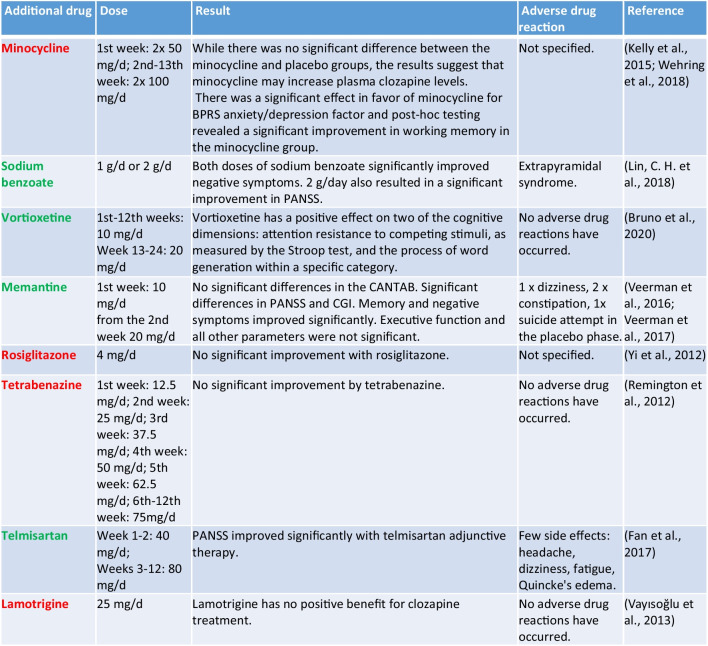
Overview of drugs in combination with clozapine (green, the drug has a positive influence on clozapine therapy; yellow, the influence on clozapine therapy is not entirely clear; red, the drug has no positive influence on therapy with clozapine)

Memantine, sodium benzoate, and telmisartan as add-on therapy improved PANSS (Fan et al. 2017; Lin et al. 2018; Veerman et al. 2017). Sodium benzoate was tested in patients with treatment-resistant schizophrenia (Lin et al. 2018). Twenty-six weeks of augmentation of clozapine with memantine in patients with refractory schizophrenia significantly improved working memory and PANSS (Veerman et al. 2016). This positive effect was also observed after 1 year of continued treatment of patients with a positive response to memantine (Veerman et al. 2017). In this study, only those participants who responded well to memantine in the previous study were included, so this study lacks a control group. The drugs marked in red in Table 4, rosiglitazone, tetrabenazine, and lamotrigine as add-on therapy to clozapine, did not show positive changes in symptoms (Remington et al. 2012; Vayısoğlu et al. 2013; Yi et al. 2012). Vortioxetine only had a positive effect on two cognitive dimensions (Bruno et al. 2020).

#### Combination of clozapine with another mGPCR-antagonist

Five mGPCR-antagonists were tested, three of which proved to be potential add-on drugs to clozapine. Sertindole and pimozide do not appear to be drugs that combine well with clozapine (Table 5) (Gunduz-Bruce et al. 2013; Nielsen et al. 2012a). The addition of ziprasidone, haloperidol, or aripiprazole to clozapine was a promising combination in patients with refractory schizophrenia (Cipriani et al. 2013; Muscatello et al. 2014). All three drugs were tested in patients with refractory schizophrenia, with ziprasidone improving negative symptoms (Muscatello et al. 2014). As an alternative to clozapine, ziprasidone was associated with very good results (Li et al. 2013). 

**Table 5:**
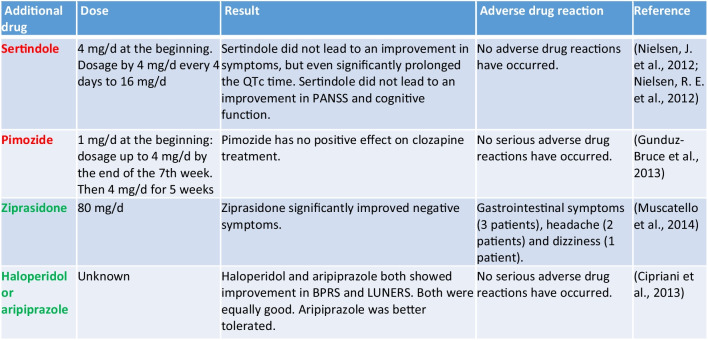
Overview of mGPCR antagonists in combination with clozapine (green, the drug has a positive influence on clozapine therapy; yellow, the influence on clozapine therapy is not entirely clear; red, the drug has no positive influence on therapy with clozapine)

#### Studies without allocation

As the drugs tested were so heterogeneous, they could not be assigned to any other group. This applies to five studies (Table 6). Patients who have intentionally or unintentionally ingested too high a dose of clozapine suffer from severe symptoms of intoxication. This is due to the numerous G-protein-coupled receptors at which clozapine acts. These symptoms include coma, confusion, seizures, and respiratory distress (https://www.apotheken-umschau.de/medikamente/beipackzettel/clozapin-hexal-25-mg-tabletten-1403645. html#:~:text=It%20can%20lead%20to%20a%20many%20low%20blood%pressure%2C%20collapse%20and%20respiratory distress; last accessed: 07.12.2023). This makes it all the more important to find possible therapies to respond to intoxication with clozapine. SMOF lipid infusion has not only led to a significant improvement in symptoms of intoxication but to a significantly shorter length of hospitalisation (Elgazzar et al. 2021; Li et al. 2013).

Vitamin D did not have a better effect on mental symptoms and metabolic parameters than placebo (Table 6). Only cognition improved, but with little significance (Krivoy et al. 2017).

In schizophrenia patients, nicotine replacement therapy with nicotine patches or a single dose of bupropion did not lead to smoking cessation. Only 18 out of 287 patients managed to quit smoking with one of the two treatment options (Wu and Lan 2017).

**Table 6:**
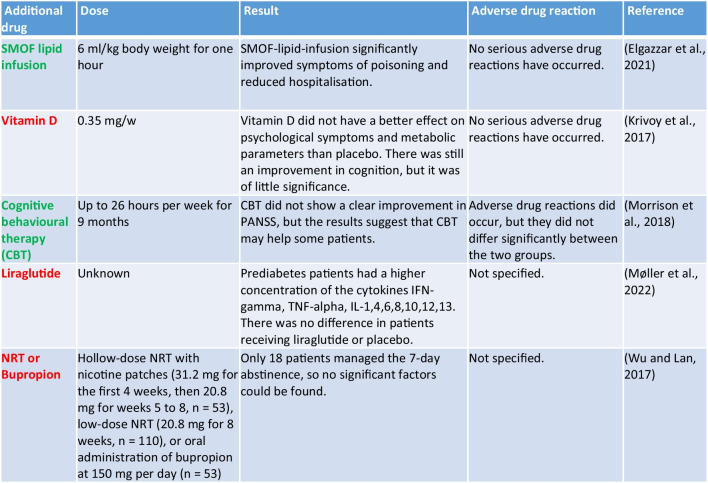
Overview of studies not assigned to a specific group (green, the drug has a positive influence on clozapine therapy; yellow, the influence on clozapine therapy is not entirely clear; red, the drug has no positive influence on therapy with clozapine)

Cognitive behavioural therapy also had no significant positive influence on clozapine therapy. However, some patients responded very well to this additional therapy, so it must be decided on an individual basis whether the patient could benefit from it (Morrison et al. 2018).

Liraglutide did not change the concentration of cytokines. Prediabetes patients showed increased levels of the cytokines IFN-gamma, TNF-alpha, and IL-1, IL-4, IL-6, IL-8, IL-10, IL-12, and IL-13 (Møller et al. 2022).

#### Comparison with reviews

The results of these studies were compared with four previous and recent reviews on clozapine (dated from 2013, 2016, 2018, and 2021). In the 2013 review, 15 different “antipsychotic” drugs, including clozapine, were analysed about their efficacy and tolerability in a total of 212 studies involving 43,049 patients. The results are similar to the results of our study. Compared to placebo, clozapine yielded the best result in terms of symptomatic improvement. Clozapine led to the strongest sedation but had the lowest risk of EPS. Differences in efficacy among mGPCR-antagonists were also observed. The conclusion of the review was that the drugs should no longer be categorised into first and second generation, but rather into hierarchies. This should make it easier for the treating physician to plan an individualised therapy (Leucht et al. 2013).

The 2016 review also found comparable results. In 21 studies, 2,364 patients with refractory schizophrenia were examined, 1,131 of whom were treated with clozapine. Clozapine was superior to other drugs in improving positive symptoms in the short and long term, as well as in improving negative and overall symptoms in the short term. In treatment-resistant schizophrenia, clozapine should be used as the treatment of first choice. However, if there is no response after 6 months, a switch to another drug should be made, as clozapine has a broad spectrum of adverse effects (Siskind et al. 2016).

The third review focussed on drugs that could enhance clozapine (46 studies, 2,223 participants). The symptoms of overall psychosis were improved by aripiprazole, fluoxetine, and sodium valproate. For negative symptoms, memantine was most effective as an augmentation of clozapine (Siskind et al. 2018b). Memantine also showed an improvement in symptoms (Veerman et al. 2017).

In a further review on 112 studies, not only the efficacy of clozapine was compared, but also adverse effects and possible additional therapies. Clozapine was superior in terms of overall and positive symptoms. For treatment-resistant positive symptoms, clozapine was more effective compared to quetiapine and haloperidol, but not compared to olanzapine. Clozapine led to unfavourable metabolic outcomes, as well as constipation. However, clozapine again showed a lower risk of triggering EPS. Metformin, GLP-1 receptor antagonists, and, to a lesser extent, aripiprazole appear to be useful adjuncts for the treatment of clozapine-induced weight gain (Wagner et al. 2021). These drugs were also found to be effective in the studies in this review.

#### Comparison of the results of this analysis with three handbooks

The results of this study were compared with the content of three handbooks (Table 7). Clozapine is predominantly used for treatment-resistant schizophrenia but the fear of agranulocytosis is so large that psychiatrists often delay the use of clozapine for too long (Meyer and Stahl 2019). Clozapine is the most effective drug for treatment resistance and administration should not be delayed if possible (Taylor et al. 2018). No drug showed a clear benefit as augmentation of clozapine. If a drug had a positive effect on clozapine therapy, it was very small. Aripiprazole, haloperidol, and ziprasidone also showed a possible benefit in the combination with clozapine in the studies included here (Cipriani et al. 2013; Muscatello et al. 2014). However, the physician should decide on an individual basis whether the patient can benefit from augmentation of clozapine. Lamotrigine was not associated with benefits (Vayısoğlu et al. 2013) as well as in the book “Kompendium der Psychiatrischen Pharmakotherapie” (Benkert and Hippius 2023). In contrast, all three books are unanimous in their opinions on the treatment of weight gain with clozapine. Metformin is the drug of choice, which can also be seen from the studies in this analysis. A total of three studies were included in this report, all of which showed good effects on weight (Chen et al. 2013; Chen et al. 2015; Chiu et al. 2016). Liraglutide, fluvoxamine, and aripiprazole are mentioned as alternatives. These three drugs also showed positive effects on weight under clozapine in the included studies (Fan et al. 2013; Larsen et al. 2017; Lu et al. 2018; Svensson et al. 2019). Different drugs are mentioned in all three books for the treatment of clozapine-induced hypersalivation. Atropine drops, metoclopramide, a sublingual ipratropium spray, and botulinum toxin type B injections dominate (Table 7). The studies on atropine drops and metoclopramide included in this report also showed good results for the treatment of hypersalivation (Kreinin et al. 2016; Mubaslat and Lambert 2020). In summary, there is not yet a clear solution for augmenting clozapine, which makes it important that new studies are conducted. In addition to the drugs mentioned above, vortioxetine, memantine, sodium benzoate, and telmisartan also yielded promising results in this analysis, which should be followed up (Bruno et al. 2020; Fan et al. 2017; Lin et al. 2018; Veerman et al. 2016; Veerman et al. 2017). There are many studies and treatment concepts for clozapine-induced weight gain and hypersalivation that could be improved in further studies.

**Table 7:** Comparison of content with three handbooks

	Compendium of psychiatric pharmacotherapy (Kompendium der Psychiatrischen Pharmakotherapie)	The Maudsley prescribing guidelines in psychiatry	The clozapine handbook: Stahl’s handbooks
Indication of clozapine	Clozapine only in case of resistance to therapy. In case of pronounced EPS or suicidal tendencies, clozapine should be used earlier (p. 309).	Clozapine is the most effective drug for refractory schizophrenia and its use should not be delayed. The use of the latest antipsychotic drugs is widespread, but not supported by research (p. 162).	The fear of the adverse effects of clozapine is so great that some psychiatrists delay clozapine therapy for refractory schizophrenia for too long, thus prolonging the suffering of patients (p. 6). Clozapine is the most effective drug for therapy resistance. In addition, clozapine works very well against suicidality and aggressive behaviour (p. 10–11).
Augmentation strategies for clozapine	Clozapine and valproate showed good results in seizure prophylaxis as well as in the treatment of depressive and affective syndromes that do not respond to clozapine (p. 387). Lamotrigine very questionable, no recommendation (p. 386).	Lamotrigine with possible effect in non-responders, aripiprazole and haloperidol with very little evidence, aripiprazole leads to weight reduction and LDL, ziprasidone in three RCTs with good results but with QTc prolongation (p. 159).	Amilsupride, aripiprazole, chlorpromazine, haloperidol, pimozide, risperidone, sertindole, and sulprides have very little effect on clozapine augmentation (p. 48).
Treatment of clozapine-induced weight gain	Metformin (p. 345–346), liraglutide (p. 346), fluvoxamine (p. 347)	First line: metformin, alternative liraglutide or aripiprazole (p. 99–100)	Metformin and aripiprazole (p. 211–212)
Treatment of clozapine-induced hypersalivation	Atropine drops sublingual, ipratropium spray sublingual, amisulpride, pirenzepine, amitriptyline, clonidine, benztropine, botulinum toxin, diphenhydramine (p. 417), and metoclopramide (pp. 352–353).	Metoclopramide with very good results, hyoscine with many adverse effects, atropine difficult to administer (p. 189).	First line: atropine drops sublingual or ipratropium oral spray; second line: botulinum toxin-B injections (p. 173).
Reference	Benkert, O. and Hippius, H.: Kompendium der Psychiatrischen Pharmakotherapie, 14th edition, Germany, Springer, 2023	Taylor, D. M., Barnes, T. R. E. and Young, A.H.: The Maudsley prescribing guidelines in psychiatry, 13th edition, England, Blackwell Pub, 2018	Meyer, J. M. and Stahl, S. M.: The clozapine handbook, 1st edition, England, Cambridge University Press, 2019

### Methodological criticism

 The quality of the studies was assessed. A simple point system was developed to place the studies into three categories (Table 8). Seven of 64 studies had rather poor methodology (Table 9). These studies were mainly CTs that, for example, looked at the effects of clozapine on the heart. Almost 40% of the studies had a good methodology, and more than 50% of the studies had a medium-quality methodology. Thus, this analysis shows that there is still a need for high-quality clinical studies on clozapine.

**Table 8:** Overview of the criteria used for the methodological critique

Methodological criteria	Definition	Reachable points
Number of participants	Less than 50 participants = 1 point, 50–100 participants = 2 points, more than 100 participants = 3 points.	3
Control group	In addition to a group of participants who take clozapine, there is another group that takes either placebo or another drug.	1
Randomisation	The participants in the study are randomly assigned to one of the groups, without any influence from the participants or evaluators.	1
Conflict of interest	None of the authors works for or receives funds from a company that would have a commercial interest in certain study results.	1
Maximum achievable score		6

**Table 9:**
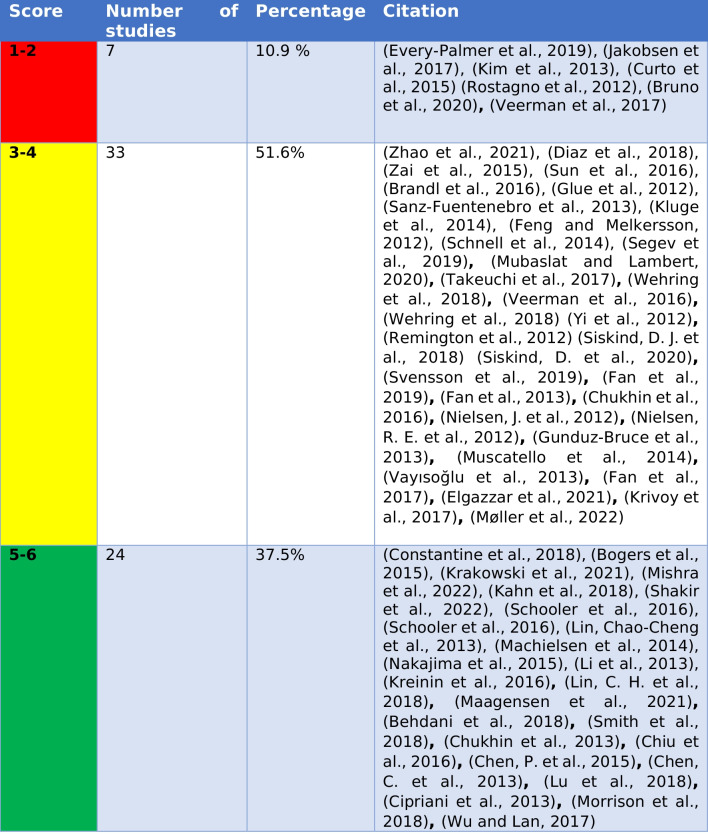
Classification of studies by quality of methodology (green, studies with a good methodology; yellow, studies with a medium methodology; red, studies with a rather poor methodology)

## Limitations

It cannot be guaranteed that all relevant studies from 2012-2022 were included. This study is intended to provide an overview of the study situation on clozapine and to evaluate which aspects should be investigated in further studies.

## Conclusions

The studies have revealed several important aspects for treatment with clozapine:

1. It is possible to switch from tablets to a suspension solution or vice versa if this seems more suitable for the patient. Plasma clozapine concentration does not change significantly when blood is collected within 10–14 h of ingestion. The type of blood collection could be important for the success of the therapy. If possible, capillary blood sampling should be used.
2. No mGPCR-antagonist proved to be significantly better than clozapine in the studies. Ziprasidone and risperidone can be used well for the treatment of schizophrenia. Ziprasidone improved symptoms in combination with clozapine, so this drug probably has potential for schizophrenia treatment, also as a possible alternative to clozapine.
3. After 8 years of observation, the metabolic parameters of the participants on clozapine were no worse than those of the participants on olanzapine. Olanzapine even seemed to lead to DM more frequently. Clozapine is not the only drug associated with numerous adverse effects.
4. Clozapine-induced hypersalivation appears to be well treatable. Three drugs have been shown to be effective, so individual adaptation to the patient is possible.
5. The same applies to the treatment of weight gain. Eight drugs showed positive effects here, although further studies would be useful in order to filter out the drugs that would not be suitable for long-term therapy.
6. Should acute intoxication with clozapine occur, SMOF lipid infusions appear to be a promising option. This treatment option should also be evaluated for the other mGPCR-antagonists.
7. There were no cases of agranulocytosis in any of the studies.

## Data Availability

All source data for this study are available upon reasonable request.

## Abbreviations

BMI: Body mass index
BPRS: Brief Psychiatric Rating Scale
CDSS: Calgary Depression Scale for Schizophrenia
CRP: C-reactive protein
CT: Clinical trial
CUD: Cannabis use disorder
DDD: Defined daily dose
EPS: Extrapyramidal motor symptoms
LUNERS: Liverpool University Neuroleptic Side-Effect Rating Scale
LVEF: Left ventricular ejection fraction
m-GPCR antagonist: Antagonist at multiple G-protein-coupled receptors
PANSS: Positive and Negative Syndrome Scale
TDM: Therapeutic drug monitoring
TESS: Treatment Emergent Symptom Scale
RLS: Restless leg syndrome
